# Reply to S. Zhang, L. Fornaro et al, and H.J. Lee et al

**DOI:** 10.1200/JCO.2016.69.1071

**Published:** 2016-08-15

**Authors:** Shukui Qin, Jin Li

**Affiliations:** Shukui Qin, People’s Liberation Army Cancer Center, Bayi Hospital of People’s Liberation Army, Nanjing, People’s Republic of China; Jin Li, Shanghai Cancer Center, Fudan University, Shanghai, People’s Republic of China

Zhang,^1^ Fornaro et al,^2^ and Lee et al^3^ have posed several questions and suggestions regarding our study^4^ on apatinib in chemotherapy-refractory gastric cancer.

According to the concept offered by the International Conference on Harmonisation’s E9 guidelines^5^ and the China Food and Drug Administration,^6^ it is acceptable to exclude from the full analysis set (FAS) any patient who did not receive at least one dose of trial medication after random assignment. Therefore, we eliminated from the FAS six patients who did not take any trial medication. This is the same approach used in a phase III trial^7^ in which 162 patients were randomly assigned and three were eliminated from the FAS as a result of not taking any trial medication.

Of the patients in our study with gastric or gastroesophageal junction adenocarcinoma, 70% had experienced gastrectomy and 75% were male. Apatinib dosage in our phase III trial was based mainly on the dosage of the previous phase II trial of apatinib in gastric cancer.^8^ The phase II trial of apatinib in breast cancer, however, was an exploratory study, including dose exploring, and all patients were female.^9^ Use of a medicine may vary in different studies and indications, such as in the two trials of bevacizumab for breast cancer and colorectal cancer.^10,11^ In addition, the tolerance dose may differ between men and women patients, although this requires further observation.

In our trial, total dosages in cycles 1, 2, and 3 of the apatinib group were 21,117.05 mg, 20,050.00 mg, and 20,371.38 mg, respectively, and the average doses were 754.2 mg, 716.0 mg, and 730.1 mg, respectively. No treatment-related death was observed throughout the trial.

We appreciate the opportunity to respond to the quality-of-life (QoL) comments mentioned by Zhang.^1^ In our trial report, at the end of the third cycle, rates of compliance for responding to the QoL questionnaire were 34.7% in the apatinib group and 7.7% in the placebo group. It was suggested that treatment with apatinib may have an effect against the deterioration of patient QoL.

Although there were some Eastern Cooperative Oncology Group performance status differences in two groups at baseline, it was not statistically significant for a randomized, double-blind trial. Thus, this would not influence the overall survival significantly.

The results of our study reported that grade 3 to 4 proteinuria and hypertension occurred in 2.3% and 4.5% of patients, respectively, in the apatinib group. In the REGARD study mentioned by Fornaro et al,^2^ grade 3 to 4 proteinuria and hypertension developed in 4% and 8% of patients, respectively, in the ramucirumab group.^12^ Generally, proteinuria and hypertension are recognized as the main characteristic adverse event (AE) of antiangiogenesis agents and have high clinical risk.

The RAINBOW^13^ trial focused on European and American populations; however, gastric carcinoma in China tends toward younger patients, that is, people age 45 to 64 years have the highest incidence.^14^ Hence, it is well founded that we enrolled patients age < 70 years in our trial.

Several experimental studies and clinical trials about the efficacy and safety of apatinib combined with chemotherapy are ongoing. Preliminary data have showed synergistic effects of combination therapy and unchanged adverse drug reaction profiles.

Cardiotoxicity-related AEs were observed, reported, and analyzed exactly in this trial. Cardiac toxicity of apatinib was atypical and most AEs were mild or moderate. There was no statistically significant difference in cardiac toxicity between the apatinib and placebo groups.

The relationship between the specific AEs and the efficiency of apatinib has been noted, as Lee et al^3^ write. In our trial, overall survival for patients who had hypertension, proteinuria, and hand-foot skin reaction was greater than that of patients who did not experience those AEs. These specific AEs could be considered surrogate clinical biomarkers of drug activity. Relevant data analysis will be published soon.

Apatinib is a small molecular and multiple-target tyrosine kinase inhibitor, whereas ramucirumab is a large molecular and humanized IgG1 monoclonal antibody. They both mainly target vascular endothelial growth factor receptor, but with different mechanisms. Ramucirumab has not yet been approved for use in China. We look forward to proceeding with head-to-head studies of the two drugs in the future.
